# Predicting seizure onset zones from interictal intracranial EEG using functional connectivity and machine learning

**DOI:** 10.1038/s41598-025-02679-4

**Published:** 2025-05-22

**Authors:** Jared Pilet, Scott A. Beardsley, Chad Carlson, Christopher T. Anderson, Candida Ustine, Sean Lew, Wade Mueller, Manoj Raghavan

**Affiliations:** 1 Joint Department of Biomedical Engineering, Marquette University and Medical College of Wisconsin, Milwaukee, WI USA; 2 Department of Neurology, Medical College of Wisconsin, Milwaukee, WI USA; 3 Department of Neurosurgery, Medical College of Wisconsin, Milwaukee, WI USA

**Keywords:** Machine learning, Intracranial EEG, Functional connectivity, Seizure onset zone, Epileptogenicity, Biomarkers, Biomedical engineering, Epilepsy, Neuroscience

## Abstract

Functional connectivity (FC) analyses of intracranial EEG (iEEG) signals can potentially improve the mapping of epileptic networks in drug-resistant focal epilepsy. However, it remains unclear whether FC-based metrics provide additional value beyond established epilepsy biomarkers such as epileptic spikes and high-frequency oscillations (HFOs). Using interictal iEEG data from 26 patients, we estimated FC across eight frequency bands (4–290 Hz) using amplitude envelope correlation (AEC) and phase locking value (PLV). From the resulting FC-matrices, we estimated two graph metrics each to derive 32 FC-based features. We also extracted features related to spikes, HFOs, and power spectral densities (PSD). A trained support vector machine (SVM) classifier predicted seizure onset zones (SOZs) with an area under the ROC curve (AUC) of 0.91 for node-level 4-fold cross-validation (CV), 0.69 for patient-level 4-fold CV, and 0.73 for patient-level leave-one-out CV. Notably, gamma-band graph features from AECs outperformed spikes and HFOs in SOZ prediction when using an equivalent number of features. Our results strongly suggest that AEC-based features may provide more information about epileptogenicity compared to PLV-based features. Furthermore, machine learning provides a robust approach for identifying useful FC-based features and integrating information from putative biomarkers of epilepsy to better localize epileptogenic networks.

**Supplementary Information:**

The online version contains supplementary material available at 10.1038/s41598-025-02679-4.

## Introduction

Epilepsy is a disease of brain networks characterized by a predisposition to recurrent seizures and the sequelae of seizures^1^. Drug-resistant epilepsy (DRE), where seizures are refractory to adequate trials of two or more anti-seizure medications occurs in a substantial proportion of patients, with estimates ranging from ~ 15% in the community to as high as 35% in clinic-based populations^2–6^. While several non-pharmacologic treatment options are now available to manage drug-resistant epilepsy^7–10^, a sizable proportion of patients with focal DRE may benefit from surgical resection or ablation of the brain region(s) that give rise to their seizures^11–13^. Invasive intracranial EEG (iEEG) studies that capture the patient’s habitual seizures are very often necessary to delineate seizure onset zones (SOZs) which provide an approximation of the minimum amount of brain tissue that must be resected or ablated to yield seizure freedom—i.e., the hypothetical epileptogenic zone (EZ)^14–17^. Over the long term, seizure recurrence is unfortunately observed in a substantial proportion of patients; this is more frequent in non-lesional and extratemporal focal epilepsy and in patients with longer duration of epilepsy^12,18–21^. The limited spatial sampling of the brain that is possible using surgically implanted subdural or depth electrode arrays is a major limitation in delineating the precise extent of the SOZ. Furthermore, resections may sometimes be limited by the presence of eloquent cortex^14^. However, seizure recurrences that are observed even when resections are deemed adequate underscore the fact that the SOZ only represents an approximation of the EZ^22^. There is thus enormous interest in interictal iEEG biomarkers that may provide better characterization of epileptogenic brain networks.

Over the last couple of decades, several putative biomarkers derived from quantitative analysis of EEG signals have been explored with varying success in predicting the EZ or surgical outcomes, including measures that quantify epileptic spike discharges^23–26^, focal slow activity^27–29^, HFOs^30–36^, phase-amplitude coupling^37–39^, and aspects of EEG dynamics^40–43^. Recently, functional connectivity (FC) has been explored by many groups to characterize epileptic networks in the brain^44–53^. While individual epilepsy biomarkers may provide a partial characterization of epileptogenic networks, the integration of information provided by different putative biomarkers could potentially yield better approximations of the EZ. There have thus far been few studies that have utilized machine learning (ML) to integrate information across multiple interictal features of intracranially recorded ECoG/SEEG signals to map seizure-generating networks^38,54–58^. In this study, we sought to determine whether integration of information derived from FC and established biomarkers of epilepsy such as epileptic spikes and HFOs using ML can help improve the mapping of the EZ. We characterized interictal FC from iEEG signals recorded before epilepsy surgery using narrow-band amplitude envelope correlations (AEC) and phase locking values (PLV). Recent work from our laboratory has shown that graph metrics derived from AECs are more robustly elevated than those derived from PLVs within SOZs, especially in the gamma frequency band^52^, but this does not exclude the possibility that the latter provides useful information about epileptogenicity. We hypothesized that the integration of information from multiple iEEG features including those derived from FC using ML will improve the identification of epileptogenic cortex compared to determinations made from any one feature of the iEEG alone. Furthermore, we hypothesized that FC-based features provide information about epileptogenicity that is additive to what can be learnt from epileptic spikes, HFOs, or power spectral characteristics of the iEEG signals. We tested these predictions by training ML models on features derived from iEEG signals with the clinically annotated SOZ and resection zones (RZ) as the targets for the classifier. Since all patients in our cohort had good seizure outcomes after surgery, we treated the SOZ as a surrogate for the EZ in our analysis. We first compared several machine-learning methods for this classification task using both K-fold and leave-one-out (LOO) CV methods to identify the models that performed the best. We then assessed the dependence of classification accuracy on the number of features available and thereafter assessed the relative performance of different feature-sets for the classification task.

## Methods

### Intracranial EEG data

This study utilized archived iEEG data from adults with drug-resistant focal epilepsy who underwent invasive iEEG studies before epilepsy surgery. The recordings were acquired between 2008 and 2018. Informed consent was obtained from all patients and/or their legal guardians. This study was performed under a protocol approved by the Institutional Review Board of the Medical College of Wisconsin (PRO15813) and all research activities were conducted in compliance with relevant guidelines and regulations. Inclusion criteria required an Engel Class 1 A seizure outcome for at least 2 years following surgery, availability of an awake sample of iEEG from the first 24 h of recording at least 1 h removed from any seizure activity, and the primary use of a subdural grid to ensure homogeneity of the dataset. Exclusion criteria included epilepsies related to high-grade tumors and perioperative strokes at the resection margin. We analyzed data from 26 patients who met al.l criteria. The clinical variables for all patients were retrieved from the medical records and have been previously published^52^.

All iEEG data samples were collected during multi-day, invasive video-EEG recordings using Nihon Kohden *Neurofax* systems (Nihon Kohden, Tokyo, Japan). Recordings were collected using either 128 or 192-channel amplifiers and subdural grid and strip electrodes. Of the 26 patients included in this study, three had depth electrodes sampling the mesial temporal structures in addition to the subdural electrodes. Subdural electrodes were constructed of platinum-iridium alloy discs 4 mm in diameter and were arranged in 8 × 8 grid, 8 × 2 partial grid, 4 × 1 strip, 6 × 1 strip, or a combination of types with 10 mm spacing (Ad-Tech Medical, Oak Creek, Wisconsin). Depth electrodes were constructed of platinum-iridium alloy on a depth probe 1.1 mm in diameter with 5 mm spacing (Ad-Tech Medical, Oak Creek, Wisconsin).

Depending on desired coverage and at the neurosurgeon’s discretion, grid corners or entire rows were removed during implantation. The iEEG signals were acquired at a sampling rate of 1000 Hz. A system reference for the recording was provided by a 2-channel electrode anchored at the craniotomy margin with titanium screws. EKG and videographic data were acquired simultaneously. To localize seizure onset zones, anticonvulsants were tapered during the recording to provoke seizures while under observation in a Neurological Intensive Care Unit. Following the capture of sufficient seizure data, patients were restarted on their regimen of anticonvulsant medications. When clinically indicated, electrical stimulation mapping was performed to identify functional cortices to guide the surgical plan for ablation or resection.

The data archived from these studies included 30-minutes each of awake and asleep interictal iEEG samples from each day of recording, segments containing clinical events or electrographic seizures, and, when performed, recordings from the stimulation mapping of the cortex. Data samples analyzed in this study consist of the first 30-minute sample of awake interictal iEEG archived from the first 24 h of recording. All iEEG data was reviewed visually by an epileptologist, and channels with excessive noise were eliminated. All records were exported as ASCII text files for further analysis using custom MATLAB (The MathWorks, Inc., Natick, Massachusetts, United States) scripts.

### SOZ and RZ maps

High-resolution CT and MRI were performed for each patient following the implantation of electrodes. Electrode locations were reconstructed using the CT-MRI co-registration methods that have been described in detail in a previous publication^59^. The imaging data for localizing electrodes and post-operative imaging data were retrieved for all patients and reviewed. The clinical reports and electrode maps relating to the iEEG studies along with the imaging data for electrode locations and post-operative resection volumes were reviewed by an epileptologist for each patient.

The designation of seizure onset zones (SOZ) was based on the clinical determinations made at the time of the patient’s surgery. No secondary review of seizure recordings was performed specifically for this study. A binary SOZ map was generated for each patient relative to their electrode array with ‘1’ meaning an electrode had been determined to be part of the SOZ. Binary resection zone (RZ) maps were constructed manually using the visualizations of intracranial electrodes from CT-MRI co-registered data along with the post-operative imaging data. Electrodes within the resection as well as 5 mm of the resection margins were assigned to the RZ given the frequent presence of gliosis at the resection margins.

### Feature extraction

#### iEEG preprocessing

The iEEG data from each patient was processed using a combination of custom MATLAB scripts, functions from *EEGLAB* (https://sccn.ucsd.edu/eeglab/), and functions from the *Fieldtrip* toolbox (http://www.fieldtriptoolbox.org/). All analyses were performed at the original sampling rate of 1000 Hz. Channels containing excessive noise or continuous artifacts were eliminated from further analysis based on visual inspection by an epileptologist. Power line noise and its first five harmonics were removed from the recordings using a spectrum estimation method^60^ as implemented in the *removeLineNoise_SpectrumEstimation* MATLAB function which is publicly available (https://www.mathworks.com/matlabcentral/fileexchange/54228-remove-line-noise). In this method, the edges of the power spectrum adjacent to the power line noise frequency and its harmonics are interpolated. We chose this method since it is less prone to produce distortions of the time domain signal compared to traditional notch filters. Although the frequency band definitions for our analysis avoid the harmonics of the power line noise, some of our bands are immediately adjacent to the frequencies of harmonics (within 10 Hz). Given the soft roll-off of the filter edges, this creates the potential for power line noise to leak into bands of interest and produce spurious AECs. We therefore felt it would be prudent to explicitly remove the line noise. The pre-processed iEEG data was then re-referenced to the common average reference. This process was repeated for all patients. Following preprocessing, a total of 2176 channels of data (ranging between 41 and 121 per patient) remained available for analysis with 344 and 622 channels belonging to the SOZ and RZ, respectively. This results in an average of 13.2 (3–45) SOZ channels and 23.9 (8–50) RZ channels per patient.

#### Automatic detection of spikes and HFOs

iEEG data were subjected to automated epileptic spike detection using a previously published method and software that has been validated by comparison to human experts^61^. High-frequency oscillations (HFOs) were detected using the MNI HFO detector^62^ as implemented in the publicly available MATLAB package HFOApp^63^ with a target HFO band of 80–250 Hz. Preliminary analysis of automated spike and HFO detection using the default detection thresholds for the detectors resulted in detections which we believed to be too lenient for our dataset. As such, the thresholds of both detectors were raised to ensure higher specificity. The specific details of the parameters changed to increase specificity are outlined in the supplementary information. Average rates and amplitudes for detected spikes and HFOs were recorded for each channel. Spike and HFOs thus yielded a total of 4 features per channel of iEEG data from each patient.

#### Power spectral density estimation

The power spectral density (PSD) of the iEEG signals in dB was estimated using Welch’s method and 2-second Hanning windows with 50% overlap. PSD was estimated for nine frequency bands (1–4 Hz, 4–8 Hz, 8–12 Hz, 15–25 Hz, 35–50 Hz, 70–110, 130–170 Hz, 190–230 Hz, and 250–290 Hz) to yield nine additional features per channel of iEEG. The selected frequency bands were chosen to ensure that the 60 Hz line noise signal and its harmonics would have as minimal of an impact on the estimated PSDs as possible.

#### Functional connectivity estimation

We estimated functional connectivity (FC) in eight frequency bands (4–8 Hz, 8–12 Hz, 15–25 Hz, 35–50 Hz, 70–110, 130–170 Hz, 190–230 Hz, and 250–290 Hz) using two different measures of FC. Continuous iEEG recordings were bandpass filtered using a two-pass (zero phase) FIR filter implemented in the *FieldTrip* function, *ft_preproc_bandpassfilter*. The filtered iEEG data was then segmented into 12-second, nonoverlapping epochs, which resulted in a total of 150 epochs per 30-minute recording. Epochs containing unfiltered signal amplitudes greater than 3000 µV on any channel were excluded. To ensure that FC was estimated using the same amount of data for all patients, 100 randomly selected epochs were used for FC estimations. Both FC measures used in this study are derived from the Hilbert transform of the iEEG signals. Before performing the Hilbert transform, iEEG epochs were mirror-padded to minimize edge effects. After estimating the Hilbert transform, the mirror padding was removed to preserve the original epoch length.

**Fig. 1:**
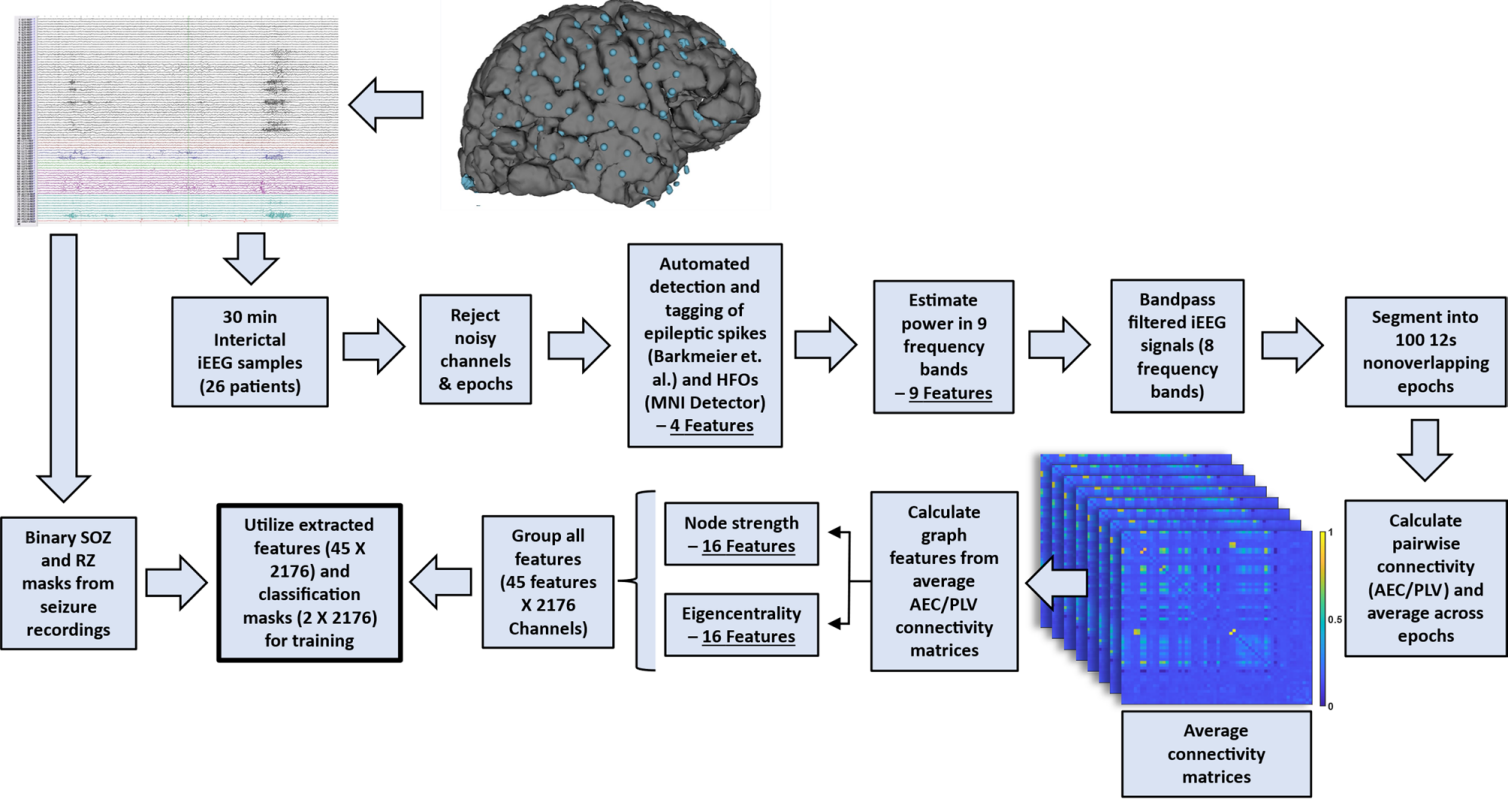
Schematic iEEG feature-extraction pipeline. Beginning with a 30-minute interictal iEEG sample, noisy channels and epochs were flagged for exclusion. Spikes and HFOs were automatically detected using the Barkmeier and MNI detectors, respectively, yielding 4 features. Power spectral density was estimated in 9 frequency bands of interest using Welch’s method yielding 9 features. The iEEG signals were bandpass filtered into 8 frequency bands of interest and segmented into 12s nonoverlapping epochs. Functional connectivity was calculated between channels in a pairwise fashion across frequency bands for each of the functional connectivity measures. Connectivity matrices were then averaged across epochs. 2 graph theoretic measures, node strength and eigencentrality, were calculated for each of the average connectivity matrices (16 total) yielding 32 features. The features were aggregated into a 45 feature by 2176 channel matrix for classifier training. Both the feature matrix and clinically defined SOZ and RZ masks (2176 element binary vectors) were utilized for classifier training. All analyses were performed in MATLAB. Details for each step are described in the methods section.

**Fig. 2:**
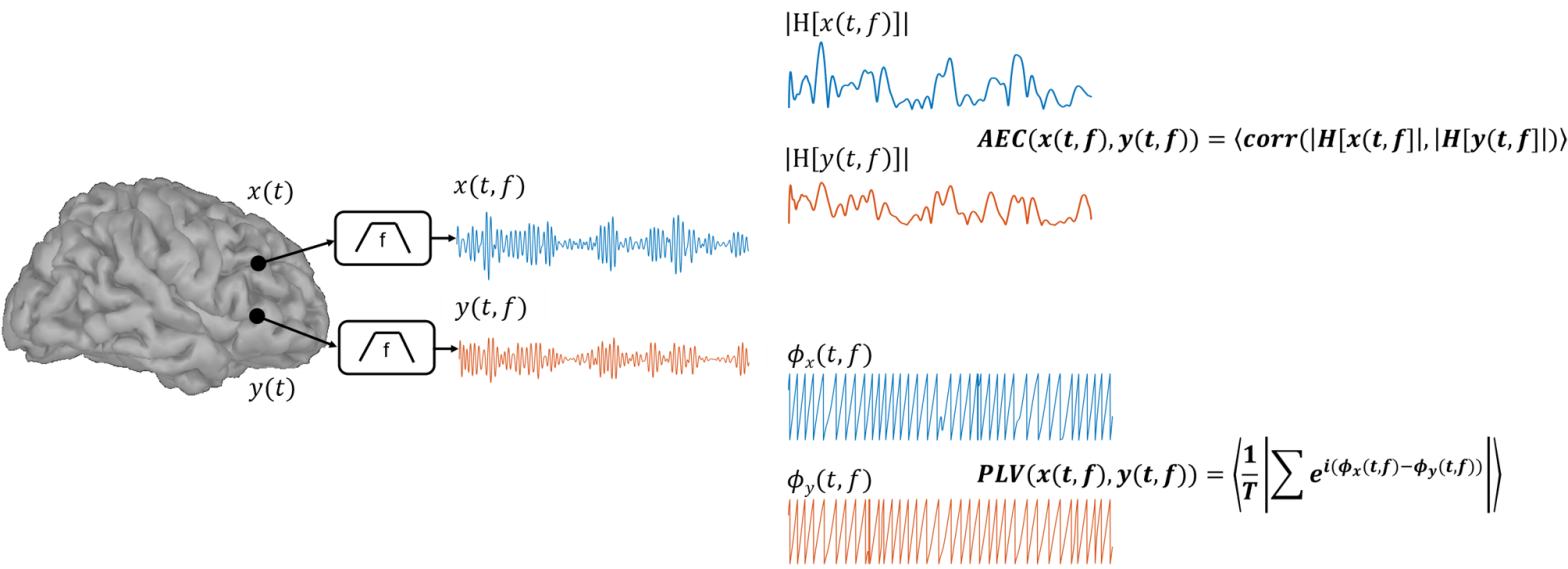
A schematic example of the pairwise functional connectivity (FC) calculations for the amplitude envelope correlation (AEC) and phase locking value (PLV). For each pair of electrodes, the time series signals, (e.g. *x*(*t*) and *y*(*t*); blue and orange, respectively) were filtered into a series of frequency bands (*x*(*t*,* f*) and *y*(*t*,* f*)) and the Hilbert transform was applied. The amplitude envelopes, |H[*x*(*t*,* f*)]| and |H[*y*(*t*,* f*)]|, were extracted by computing the magnitude of the Hilbert transformed signal. The AEC was calculated from the correlation between the two amplitude envelopes. The instantaneous phase, *ϕ*_x_(*t*,* f*) and *ϕ*_y_(*t*,* f*), was estimated by computing the phase angle of the Hilbert transformed signal. The PLV was calculated from the absolute value of the mean phase differences between the two signals.

The amplitude-envelope and instantaneous phase were calculated from the Hilbert-transforms of the iEEG signals. The amplitude-envelope correlations (AECs) for any given epoch are the Pearson correlation coefficients of the envelopes for all channel pairs. These values were then averaged across epochs to yield an average AEC for each pair of channels. Similarly, phase-locking values (PLV) were calculated from the Hilbert-transformed iEEG signals using the definition for resting state PLV^64–66^ and averaged across epochs. From the connectivity matrices estimated using AECs and PLVs in different frequency bands for each patient, two graph-theoretic metrics, node strength, and eigenvector centrality, were calculated for each patient using the Brain Connectivity Toolbox^67^. No thresholding of the FC values was performed before estimating the graph metrics. Node strength values were normalized by the number of iEEG channels for a given patient to account for differences across patients. All calculated graph features were rescaled to a 0–1 range based on the maximum and minimum values at the individual patient level. FC analysis thus yielded a total of 32 features per channel of iEEG data. Figure 1 outlines the feature extraction process and Fig. 2 shows the FC estimation process. The 45 features (2 spike features, 2 HFO features, 9 PSD features, and 32 FC features) were concatenated to yield a final structure of 45 features X 2176 iEEG channels for training and evaluating classifiers.

### Machine learning for SOZ and RZ prediction

**Fig. 3:**
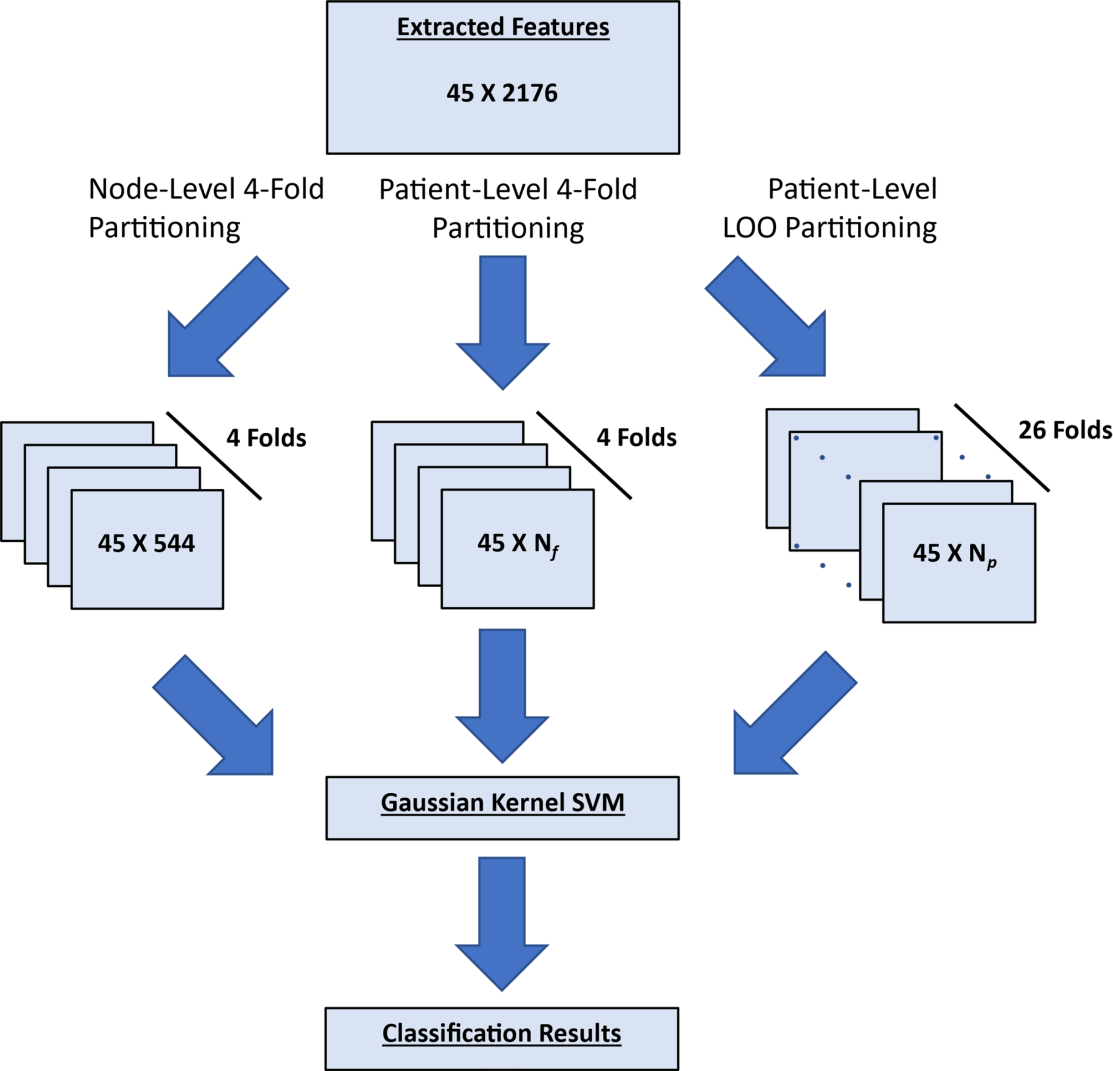
Classifier training pipeline for node-level 4-fold, patient-level 4-fold, and patient-level leave-one-out (LOO) cross-validation (CV). For the patient-level 4-fold CV, N_f_ = the total number of nodes for patients in the test fold (421–661 for our analysis). For the patient-level LOO CV, N_p_ = the number of nodes for the test patient (41–121). In all cases, the total number of nodes used across the training and test sets is equal to 2176. The patient-level 4-fold and node-level 4-fold cross-validations were repeated 10 times with different partitions for each repetition.

ML models were trained with prediction of either the SOZ or RZ as the classification objective. Prior to classifier training, feature vectors were rescaled by the maximum value of the feature across all patients so that magnitude variations across features were minimized while preserving the rank order of any given feature across channels in any given patient. Three methods of cross-validation (CV) were used: node-level 4-fold, patient-level 4-fold, and a patient-level leave-one-out (LOO). In the node-level 4-fold CV, nodes from all patients were randomly assigned to the training or test sets. For patient-level 4-fold and patient-level leave-one-out cross validations, the nodes from a given patient are either completely within the training set or the test set, with no crossover. Unlike the node-level 4-fold cross-validation, these latter two approaches simulate the situation where classifier training is based on retrospective patient data, and the testing occurs on prospective patient data. Because our dataset contains 26 patients, the patient-level 4-fold partitioning cannot be implemented exactly. The dataset was partitioned into four folds, two with 6 patients and two with 7. To improve generalizability and reduce the influence of any one partition, we repeated both the patient-level 4-fold and node-level 4-fold cross-validations 10 times with different partitions for each repetition. The partitioning of the data for these methods is outlined in Fig. 3. Classification performance was first evaluated separately for predicting the SOZ and RZ using 6 different ML methods and the node-level 4-fold CV (Fig. S1). All models were trained using a Bayesian optimization algorithm with the K-Fold loss being minimized to determine optimum model parameters over 300 iterations. To understand the characteristics of classifier performance across differing feature sets, the three best performing methods from this analysis were each evaluated using 150 different, random samples of the available 45 features. The objective was to gain insight not only into the consistency of the selected classifiers with reduced feature sets, but also the variability of classifier performance with reduced feature sets. Feature subsets with low feature-count but high SOZ classification performance may be inferred to carry more information about epileptogenicity.

A series of comparisons of classifier performance were made for different feature subsets using a gaussian kernel support vector machine (SVM). Feature subsets were selected to compare performance differences between graph measures derived from high gamma band (70–110 Hz, 130–170 Hz, 190–230 Hz) and low frequency (4–8 Hz, 8–12 Hz, 15–25 Hz) FC, differences between individual graph measures derived from the same FC measure, and comparisons between graph measures and PSD, Spike, and HFO measures. Each analysis was performed for 10 repetitions and the average AUC across repetitions was saved. Comparisons were performed for both SOZ and RZ classification using all three CV methods. All significance was determined using a paired sample Wilcoxon signed rank test at an alpha level of 0.05 using a Bonferroni correction for multiple comparisons. Paired samples are the median AUC value across CV folds from each of the 10 repetitions.

We also assessed the influence of the number of epochs used to extract features on classifier performance. For this analysis, SVM models were trained using all 45 features calculated from 6, 12, 25, 50, or 100 randomly selected epochs. For each epoch count, five random selections were analyzed. All models were trained using the node-level 4-fold CV. To ensure comparability, the channels were partitioned in the same manner for CV. This process was repeated five different times, and the mean and standard error across repetitions were calculated. These values are presented in supplemental Fig. S2. We also determined whether false positives from a trained SOZ classifier are disproportionately represented within the surrounding RZ (in the vicinity of the SOZ), as opposed to distant sites. To do so, we first established the number of false positives (and the corresponding percentage value) that were observed to fall within the RZ (but outside the SOZ) at the optimal operating point (OOP) of the ROC curve of a trained SVM classifier. The OOP was defined as the intersection between the ROC curve and a line from (0,1) to (1,0) on the same axes. We generated a permutation test distribution by randomly picking an equivalent number of nodes 5000 times from among the non-SOZ channels and determined what percentage of the channels fell within the RZ (but outside the SOZ). The percentage of the true SOZ false positives was then compared to the generated distribution using a z-test (Fig. S3).

## Results

### Patient demographics and clinical variables

The patient dataset used for this analysis consisted of 26 patients (14 M; 12 F) with an average age of 16.7 (± 10.1) years at seizure onset and 35.5 (± 12.1) years at time of surgery. The average duration of follow-up was 7.5 (± 3.1) years. MRI abnormalities were present in 17 patients (malformations of cortical development in 5; mesial temporal sclerosis in 4; low-grade gliomas in 3; vascular malformation in 3; encephalomalacia in 2). The SOZ and resections were lateralized to the left hemisphere in 14 patients. SOZs and subsequent surgical resections were in the temporal lobe in 19 patients, temporal-plus in 4, and extratemporal in 3. All patients were seizure-free (Engel Class IA) for at least 2 years following surgery. As noted earlier in the Methods section, detailed clinical variables for this patient cohort have been recently published elsewhere^52^.

### Group-level feature comparisons

**Fig. 4:**
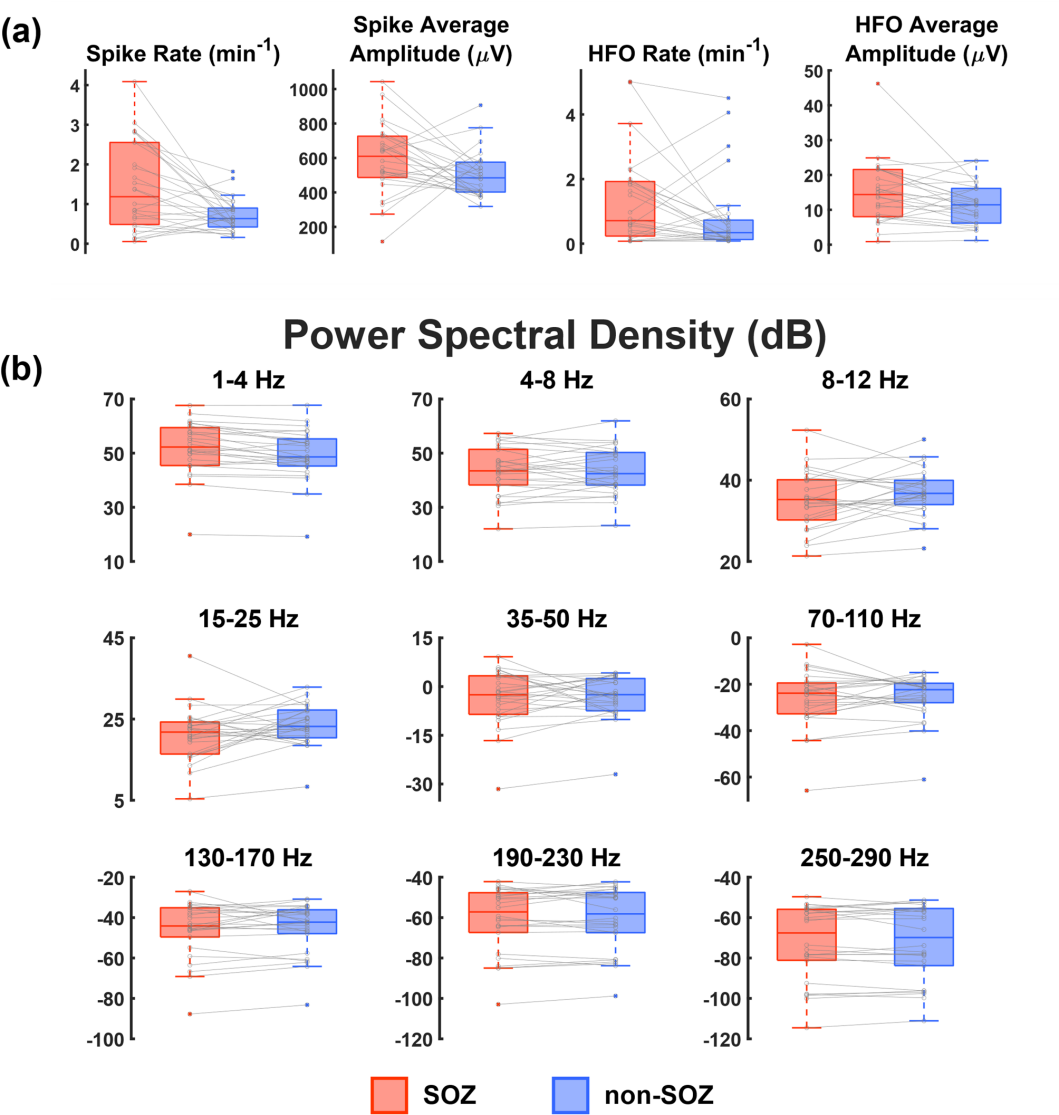
Comparisons of mean feature values across patients for (**a**) spike and HFO features and (**b**) power spectral density features. Box and whisker plots compare the aggregate values for SOZ (red) vs. non-SOZ (blue) channels. Grey markers and lines show mean values and trends for individual patients. Asterisks denote group level significance (Paired sample Wilcoxon signed rank test, α = 0.05, Bonferroni corrected across 45 features).

**Fig. 5:**
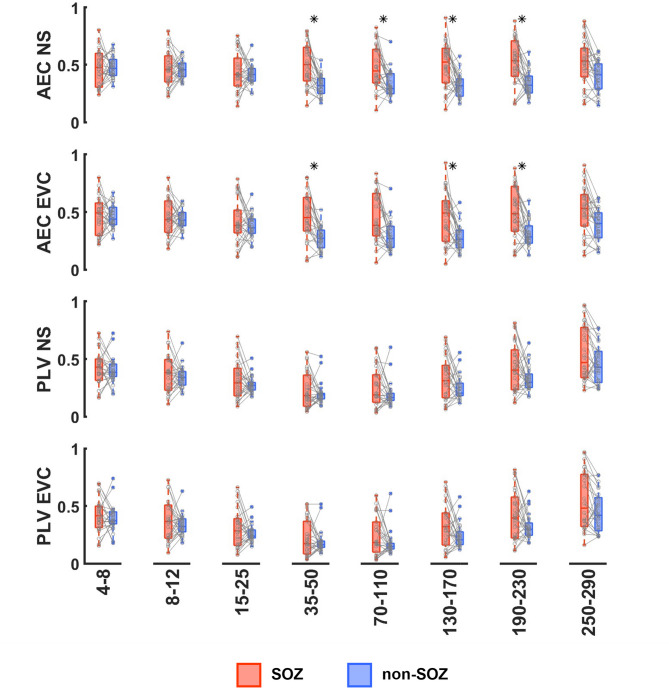
Comparisons of mean graph metric values across patients. Box and whisker plots compare the aggregate values for SOZ (red) vs. non-SOZ (blue) channels. Grey markers and lines show mean values and trends for individual patients. Asterisks denote group level significance (Paired sample Wilcoxon signed rank test, α = 0.05, Bonferroni corrected across 45 features).

Average values of FC-derived graph features for SOZ and non-SOZ channels are compared for individual patients and across the cohort in Figs. 4 and 5. Across the group, the differences between SOZ and non-SOZ channels for either spike or HFO derived features did not reach significance (Fig. 4a). Across patients, there were also no significant differences in PSD measures between SOZ and non-SOZ nodes (Fig. 4b). As shown in Fig. 5, there were no significant differences between SOZ and non-SOZ nodes at the group level for PLV-based features. Similarly, there were no significant differences at the group level for AEC-based features in the low frequency (4–8 Hz, 8–12 Hz, 15–25 Hz) bands. However, AEC-based NS was significantly higher in SOZ nodes (*p* < 0.05, Bonferroni corrected) across all gamma band frequencies (35–50 Hz, 70–110 Hz, 130–170 Hz, 190–230 Hz), and AEC-based EVC was significantly higher in SOZ nodes in most gamma band frequencies (35–50 Hz, 130–170 Hz, 190–230 Hz).

### Classifier comparisons

**Fig. 6:**
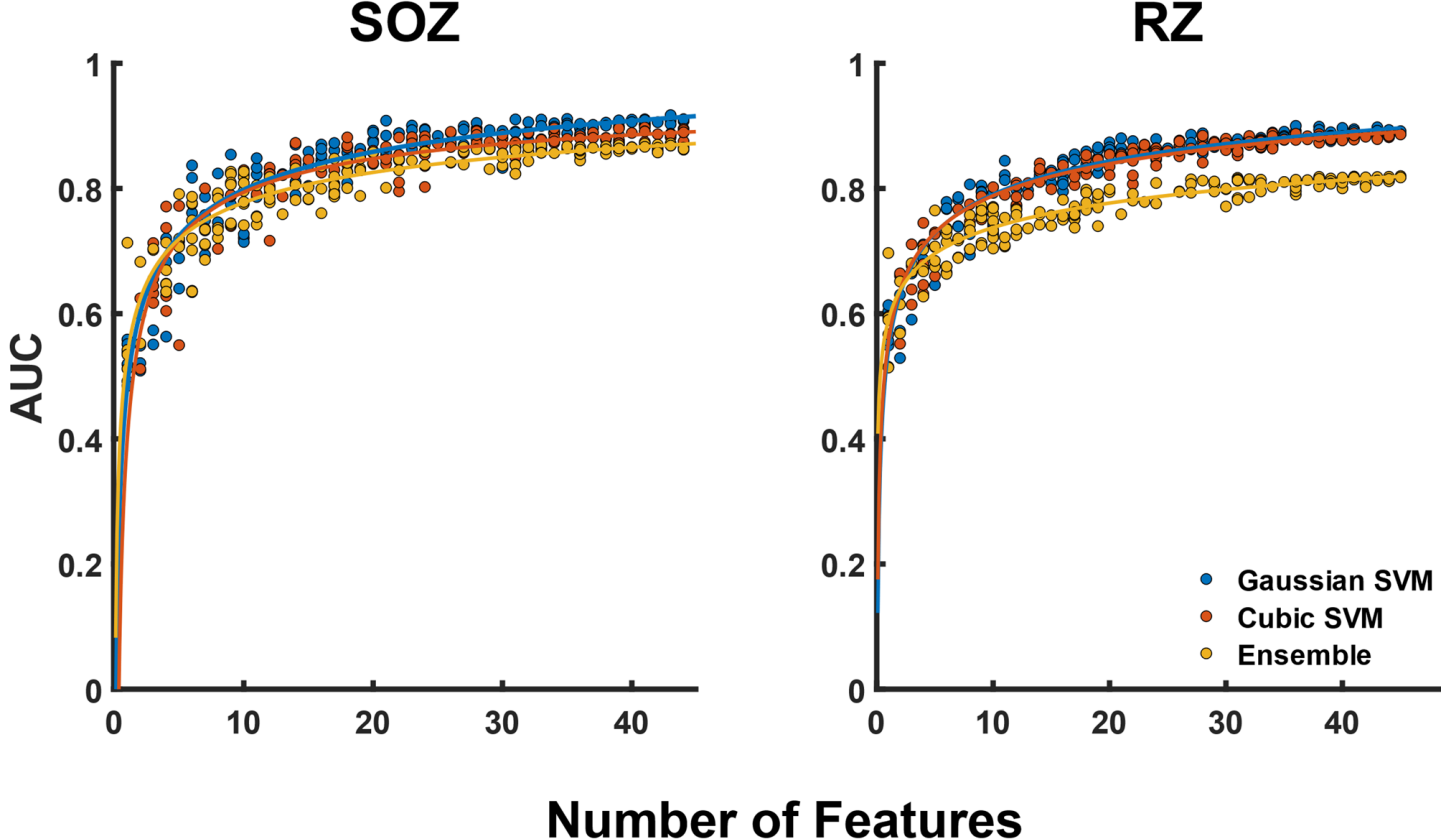
Performance comparisons for different random feature selections for both SOZ and RZ prediction. The results were evaluated using a power fit to estimate an expected AUC value for a given number of features. Points represent individual AUC values and solid lines represent the corresponding power fit for the three types of classifiers tested.

We compared six different ML models trained using all 45 available features and node-level 4-fold CV. For both types of classification tasks (SOZ and RZ), a support vector machine (SVM) using a gaussian kernel yielded the highest performance with AUCs of 0.91 and 0.90 for SOZ and RZ, respectively (Fig. S1). There was significant overlap in performance for the best classifiers, so the top three (Gaussian SVM, Ensemble, and Cubic SVM, respectively) were selected for further evaluation. Figure 6 shows how the performance of the three selected classifiers varies as a function of feature count, using different numbers of randomly selected features. Unsurprisingly, the performance of each classifier increased as the number of features used for training increased. Also, performance of all three classifiers started to plateau around ten features for SOZ and RZ classification. Whether classifying SOZ or RZ, the gaussian SVM had the highest terminal performance. While all classifiers exhibit a large degree of performance variability with reduced feature counts, the gaussian SVM consistently showed the highest performance with reduced feature counts as well. As such, the gaussian SVM was used for comparisons across targeted selections of feature subsets.

### Impact of feature set and cross-validation method on classification performance

**Fig. 7:**
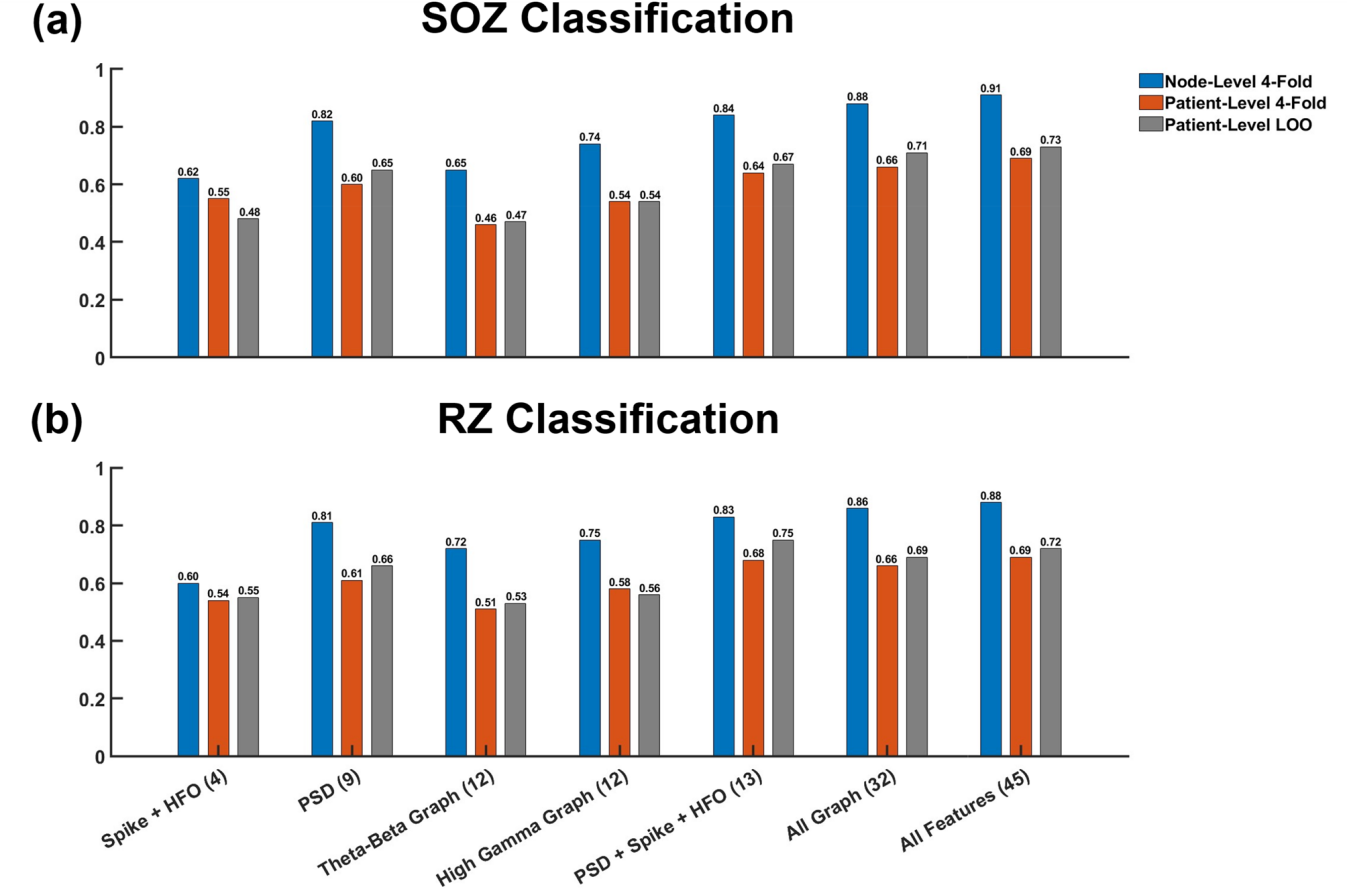
AUC comparisons for different feature sets using node-level 4-fold, patient-level 4-fold, and patient- level LOO cross-validation for predicting (**a**) SOZ and (**b**) RZ.

SOZ and RZ classifier performance using different feature subsets with node-level 4-fold, patient-level 4-fold, and patient-level LOO CV are shown in Fig. 7. As might be expected from the results shown in Fig. 6, there is a positive correlation between feature count and classifier performance regardless of CV method. For both SOZ and RZ classification, the subset of all FC derived graph features had performance closest to the ‘all features’ classifier for node-level 4-fold CV and had comparable performance in LOO CV for both SOZ and RZ classification. Specific performance comparisons between feature sets are shown in Table 1. SOZ nodes are classified more effectively by.

**Table 1:** Comparisons of classifier performance for selected pairs features derived from FC.

Feature Set	Feature Count	Node Level 4-Fold Average AUC	Corrected *p*-value	Patient Level 4-Fold Average AUC	Corrected *p*-value	Patient Level LOO Average AUC	Corrected *p*-value
All AEC	16	0.8395	**0.016**	0.6499	**0.016**	0.6544	**0.016**
All PLV		0.7026		0.5170		0.5528	
High Gamma AEC	6	0.6935	**0.016**	0.5593	0.094	0.5558	**0.016**
Theta-Beta AEC		0.5963		0.5209		0.5118	
Gamma AEC NS	4	0.7247	**0.016**	0.6193	**0.031**	0.5504	**0.016**
Spike + HFO		0.6159		0.5478		0.4759	
Gamma AEC EVC	4	0.7427	**0.016**	0.6186	**0.031**	0.5549	**0.016**
Spike + HFO		0.6159		0.5478		0.4759	

Comparisons were chosen to highlight performance differences between AEC and PLV derived graph features, performance differences between graph features derived from high frequency and low frequency FC, and performance differences between gamma AEC derived graph features versus Spike and HFO rates and amplitudes. Significance was determined using a paired-sample Wilcoxon signed rank test (α = 0.05, bonferroni corrected).

Significant values are given in bold.

AEC derived graph measures compared to those derived from PLV for all CV conditions (corrected p-values of 0.016, 0.016 and 0.016 for node-level 4-fold, patient-level 4-fold, and patient-level LOO CV respectively). Features extracted from high gamma (70–110 Hz, 130–170 Hz, 190–230 Hz) AECs outperform those from low frequency (4–8 Hz, 8–12 Hz, 15–25 Hz) AECs for node-level 4-fold and patient-level LOO CV but not for patient-level 4-fold CV (corrected p-value = 0.016, 0.094, and 0.016 for node-level 4-fold, patient-level 4-fold, and patient-level LOO CV respectively). Significance was determined using paired-sample Wilcoxon tests where the samples were median AUC values from the training repetitions.

### Sample duration for stable FC estimation

The results reported thus far utilized 100 of the available 150 epochs (each of 12 s duration) for FC estimation. This was done to ensure a consistent amount of data for analysis across all patients in the event epochs were excluded during pre-processing. The effect of the number of epochs that were averaged to extract FC measures on classifier performance (Fig. S2) suggests that performance begins to plateau at around 100 epochs. Interestingly, FC estimated from all 150 epochs were well approximated (r^2^ > 0.95) by FC’s calculated using as few as 25 and 15 epochs for AEC and PLV, respectively (Fig. S4).

## Discussion

While many putative biomarkers of epilepsy have shown promise for predicting seizure-initiating areas (SOZs) or as independent predictors of seizure outcome following epilepsy surgery, it is unlikely that any one measure alone will yield sufficiently high sensitivity and specificity for mapping the epileptogenic zone (EZ) — i.e., the minimum amount of tissue that must be removed to yield enduring seizure freedom. Recently, studies have turned to functional connectivity (FC) measures to determine if they can help improve the sensitivity and specificity of EZ prediction. Many measures, both directed and undirected, have been defined for estimating FC from physiological signals^68,69^. From FC estimates based on any one of these measures, many graph-metrics can be derived to characterize underlying brain networks. In this preliminary analysis of the relevance of FC to the mapping of epileptogenic networks, we focused on two undirected measures of FC: amplitude envelope correlation (AEC) and phase locking value (PLV). We do not assume that results based on these undirected measures will generalize to other FC measures. Our choice of AEC was based on the body of evidence that gamma-band iEEG power is strongly correlated to the firing rates of local neuron populations^70–73^, and thus AECs in the gamma-band may provide an FC measure that captures correlations in local neuronal firing rates within epileptic networks^52^. By contrast, PLV is representative of a popular class of FC measures that depend purely on narrow-band phase with no dependence on the amplitude envelopes. Using either of these measures, FC can be estimated in multiple frequency bands. Furthermore, FC estimated using any given method in any given frequency band yields multiple graph metrics that are attributable to the nodes of the network.

The problem of integrating information from many features derived from iEEG signals is especially relevant for the utilization of functional connectivity (FC) to map epileptic networks. ML not only provides a robust approach for doing so but can also be used to determine the relative value of different features and eliminate those that are of little value or redundant. Some recent studies have shown promising results for predicting the SOZ (as a surrogate for the EZ) using ML applied to features of the interictal data^54,55,58,74^.

In this study we applied ML to predict the SOZ or RZ using graph metrics derived from two different FC measures in 8 frequency bands along with more traditional measures related to epileptic spike activity, HFOs, and power spectral densities. FC was estimated using amplitude envelope correlations (AEC) and phase-locking values (PLV). The classification results for both SOZ and RZ presented in Fig. 7 demonstrate that SOZs and RZs (as surrogates for the EZ) can be predicted from interictal iEEG features with high accuracy when the 4-fold CV is done at the node-level (AUC of 0.91 and 0.88 for SOZ and RZ classification using all features), but the AUCs drop when data from any given patient is constrained to either the training or test fold (AUC of 0.69 with patient-level 4-fold CV, and 0.73 with patient-level LOO CV). This decrease in performance likely reflects limitations in classifier training occurring due to both the limited cohort size and selection of features used for training. The number of features used for the classification also had a strong influence on prediction accuracy. This is not surprising: increasing the dimensionality of the data increases the separability of the two classes of data (SOZ vs. non-SOZ) as demonstrated earlier in Fig. 6.

The marked variability in classification performance at different feature-counts suggests that feature sets carry differing amounts of information relevant to SOZ/RZ classification. To evaluate this, we compared classifier performance for different feature sets while controlling for the number of features. The utility of the FC-derived graph features was assessed by comparison to more established interictal features such as the average rates and amplitudes of interictal spikes and HFOs. Gamma-band AEC-derived features (either NS or EVCs) outperformed an equivalent number of spike and HFO related features (4 features) for classification of SOZs using node-level or patient-level 4-fold CV, and patient-level LOO CV (Table 1, bottom three rows). Additionally, when gamma-band AEC features were combined with PSD features, performance was greater than that observed when PSD features were combined with spike and HFO features for the node-level 4-fold CV (AUCs of 0.88, 0.89, and 0.84 for PSD + NS, PSD + EVC, and PSD + Spike + HFO respectively) and patient-level LOO CV (AUCs of 0.72, 0.71, and 0.67 for PSD + NS, PSD + EVC, and PSD + Spike + HFO respectively). Performance obtained using only AEC graph features was comparable to that obtained with the combination of PSD, spike, and HFO features even though only 8 graph features were used compared to 13 non-graph features for both node-level 4-fold CV (AUCs of 0.81, 0.82, 0.84 for NS, EVC, and PSD + Spike + HFO respectively) and patient-level LOO CV (AUCs of 0.67, 0.65, 0.67 for NS, EVC, and PSD + Spike + HFO respectively). The high classification performance obtained with AEC-based measures may indicate that AECs provide an indirect marker of cortical hyperexcitability^52^. However, the decreased performance obtained when performing patient-level LOO CV (0.82 vs. 0.65 for EVC) underscores the need for larger training datasets to make robust predictions of the SOZ from interictal data for individual patients.

The SOZ classification performance obtained using PLV-derived graph features was lower than that obtained using AEC-derived features (AUCs of 0.82 and 0.67 for AEC EVC and PLV EVC respectively, for instance). Some recent studies have reported better success in SOZ identification using phase-based connectivity during the transitions from interictal to ictal states^75–77^.

In this study, we used a gaussian kernel SVM to compare SOZ classification performance between selected feature sets. The determination to use this classifier was supported by the results shown in Fig. S3 and Fig. 6. Previous studies have explored the differences in performance between classifiers for a given set of feature data and have also seen improved performance from a gaussian kernel SVM relative to other classifiers^38,54^. This suggests that a gaussian kernel SVM may be a reasonable choice for classification of iEEG derived features. Additionally, this suggests that there is a certain degree of radial symmetry in the structure of metrics derived from regions of epileptogenicity which a gaussian kernel SVM is uniquely suited to capture.

To assess whether the outputs of a model trained to classify SOZs yields a measure of epileptogenicity beyond the clinically annotated SOZ, we examined whether false positives (i.e. incorrect ‘SOZ’ labels) of the classifier tend to be disproportionately represented within the RZ. Ultimately, it is the tissue included within the RZ that determines seizure outcomes. We find that this is indeed true for our trained classifiers: at the optimal operating point of the classifier (defined as the intersection of the ROC curve with the diagonal from [0,1] to [1,0]), the SOZ false-positives were disproportionately within the RZ when compared to chance (31.8% vs. 16.2%; Z = 4.028; *p* = 5.62E-5; Fig. S3). This suggests that the output of a classifier trained on a large dataset of interictal features could potentially be used to generate spatial maps of epileptogenicity, analogous to the epileptogenicity index based on ictal data that has been proposed by Bartolomei et al.^78^, but with the added ability to integrate information from multiple putative biomarkers without explicitly defining the relative weights of different features a priori. This approach would require systematic evaluation of the many potential epilepsy biomarkers based on large datasets representing a variety of surgical outcomes and epilepsy localizations, using both ECoG and SEEG recordings. Feature selection methods may then be applied to identify which features or feature families carry the most information about epileptogenicity. A future clinical tool using this approach would conceivably have two elements: a classifier that has been trained on the most useful iEEG features from a very large patient cohort, and a feature extraction pipeline that estimates these features from iEEG data from a given patient. The output of the classifier would yield a prediction for the EZ based on the most valuable features.

A previous study by Varatharajah et al.^54^, showed that the use of progressively longer interictal recordings yielded improvements in classifier performance up to durations of ~ 90 min. Our results also show a benefit from longer samples of recording, but saturation of performance appeared to occur by ~ 30 min using our collection of iEEG features, as shown in supplemental Fig. S2. This may have to do with stabilization of connectivity derived features occurring over a shorter time scale. It is conceivable that the use of multiple short-duration (~ 30 min) recordings that are separated in time may improve the performance of SOZ classification since many interictal iEEG features, including connectivity, can change over time in relation to physiological states^35^ and withdrawal of anticonvulsants for seizure provocation.

The total number of electrodes used in an iEEG study is typically much larger than the number of electrodes belonging to the SOZ. This can result in an imbalanced dataset when used to train a classifier. To account for this in our analysis, an imbalanced cost matrix was used during classifier training. This allowed classifier performance to be biased toward false positives rather than false negatives. Alternatively, one could apply different weights to the training data to bias classification towards the relevant (SOZ) channels^38^. Another popular strategy to account for imbalances within a dataset that can be applied after training is the use of precision-recall (PR) analysis. Specific comparisons between different classification performance measures including ROC and PR analyses are discussed in Hrtonova et al.^79^.

One limitation of this study is the limited sample-size of the dataset. The differences in classifier performance between the two 4-fold CV methods that we implemented may be indicative of overfitting in the node-level 4-fold implementation. This also may indicate that there is a degree of spatial information between features derived from a given patient which is being learned by the classifier in the node-level 4-fold implementation. However, the similarity in performance trends across CV methods suggests that the features do provide information about epileptogenicity. Ultimately, a larger dataset is necessary for future analysis and may decrease the observed performance differences between CV approaches. Because of the differences in performance between CV approaches, a correlation analysis was performed to determine whether characteristics associated with individual patients contributed to overall classification performance in a predictable manner. There were no strong correlations between patient-level LOO performance across feature sets and the number of channels (r^2^ = -0.024 ± 0.05), number of SOZ channels (r^2^ = -0.151 ± 0.037), or ratio of SOZ to non-SOZ channels (r^2^ = -0.185 ± 0.045). This confirms that the LOO results were not skewed by characteristics of the datasets, and that the use of a larger dataset should improve LOO classification performance.

The dataset used in this study consisted of patients who all had excellent seizure outcomes after surgery. Given the small sample size of the study, we felt that this was appropriate to be able to determine if FC-based measures carry useful information about epileptogenicity. Additionally, most patients within our cohort (19 of 26) had resections in the temporal lobes. This included patients with both mesial and neocortical temporal lobe epilepsies. Resections were temporal-plus in four (adjacent frontal, parietal, or occipital cortices) and exclusively extratemporal in only three patients. This temporal lobe bias in the data is not unusual and is consistent with the distribution of epilepsy localizations seen in typical surgical series^80–82^. It is conceivable that differences in the characteristics of iEEG signals and how they are modified by epilepsy differ between brain regions. A direction of future study, with a much larger sample size, would be to perform similar analyses using patients with a wide variety of lobar epilepsy localizations to determine if results generalize across lobes. This will allow for greater understanding of the generalizability of the approach described here, and prediction of surgical outcomes can be used to identify FC-based features that are better markers of epileptogenicity. Another important aspect of our dataset is that it is comprised primarily of subdural recordings from grid and strip electrodes, although three patients also had depth-electrodes sampling the mesial temporal structures. We acknowledge that clinical practice, including that at our epilepsy center, has shifted to primarily stereo-EEG (SEEG) studies using depth electrodes. While there are complexities associated with network construction and interpretation when combining different contact types (surface vs. depth, etc.), it does not appear that these complexities had any effect on classification performance in this study. We see no reason why the approach taken by this study cannot be replicated using SEEG recordings.

One question that is raised by our results is how to optimally utilize features when there are many possible FC-based features, or other putative biomarkers of epilepsy, that can be estimated from any given dataset. There are several feature selection strategies that can be applied to identify irrelevant or redundant features^83^. Another approach of particular interest to address this problem is that of the explainable-AI (xAI) model. This approach allows for an understanding of which features were deemed to carry high importance by a trained classifier while reducing the need for an exhaustive search of all extracted feature permutations^84^. A recent example of this with application to the identification of epileptogenic brain regions was demonstrated by Hoogteijling et al.^85^. In this study, xAI was used to determine the relative contributions of spectral features from intraoperatively recorded ECoG signals for determining whether the recorded region was resected. While these approaches may help determine which signal characteristics carry relevant information, it will be important to consider the neurophysiological mechanisms underlying the feature, which may make some features more relevant than others. Additionally, consideration should be given to the type of classifier used since this greatly impacts the types of analyses which can be performed to make an AI model explainable.

In summary, our results indicate that graph-theoretic measures derived from interictal FC estimates contain valuable information relevant to epileptogenicity. This information is maximal in the gamma band for both AEC and PLV based measures of functional connectivity. We also demonstrate that ML not only provides a powerful approach for integrating the information provided by multiple features of iEEG signals to map epileptogenicity but can also help identify those that are most useful. The general approach of using machine learning to integrate information from multiple features of brain signals to predict the EZ can potentially be applied to cortical source estimates derived from scalp EEG (especially high-density EEG) and magnetoencephalographic (MEG) recordings. While functional connectivity using MEG sources has been shown by a few recent studies to have value in predicting the SOZ and seizure-outcomes^86–88^, integration of multiple features of EEG/MEG source signals to predict the EZ remains to be explored. Application of this approach to HD-EEG or MEG sources has the potential to yield whole-brain maps of epileptogenicity in contrast to maps with limited spatial sampling that iEEG studies provide.

## Electronic supplementary material

Below is the link to the electronic supplementary material.

Supplementary Material 1

## Data Availability

Since this was a retrospective study, written informed consent that permits data to be shared publicly was not available from our patient cohort. As such, imaging and iEEG data for this study cannot be publicly shared. They may however be sought with the necessary deidentification from the senior author (M.R., mraghavan@mcw.edu), within the framework of a data-sharing agreement upon reasonable request.
