# Supplementary material for: Predicting seizure onset zones from interictal intracranial EEG using functional connectivity and machine learning
Source: Sci Rep. 2025 May 22;15:17801. doi: 10.1038/s41598-025-02679-4 (PMC12098728; doi:10.1038/s41598-025-02679-4)
Supplement: Supplementary file 1 — Supplementary Material 1 [file 41598_2025_2679_MOESM1_ESM.pdf]

Predicting seizure onset zones from interictal intracranial EEG using functional connectivity and machine learning

Supplementary Material

Automatic epileptic spike and HFO detection

As mentioned in the methods section, automated detection of epileptic spikes and high frequency oscillations (HFOs) was performed using the detector described in Barkmeier et al (2012) and the MNI detector (Zelmann et al 2012) for spikes and HFOs, respectively. Initial analysis was conducted using the default settings of the detectors. The results obtained using the default settings suggested that average amplitudes for epileptic spikes and HFOs were higher in areas clinically labelled as seizure onset zones (SOZs), but that the average rates of spikes and HFOs in these areas were decreased relative to other brain areas. This result was surprising to us, and as such, we repeated the analysis with more stringent detection criteria. For the Barkmeier spike detector, the '*STDCoeff*' variable was increased to a value of 8.5 from the default of 4. For the MNI HFO detector, the '*bs_thresh*' variable was increased to a value of 0.85 from the default of 0.67. These changes resulted in a higher specificity for both detectors. The performance of the detectors with their changes is reflected in figure 4.

Supplementary figures

Figure S1

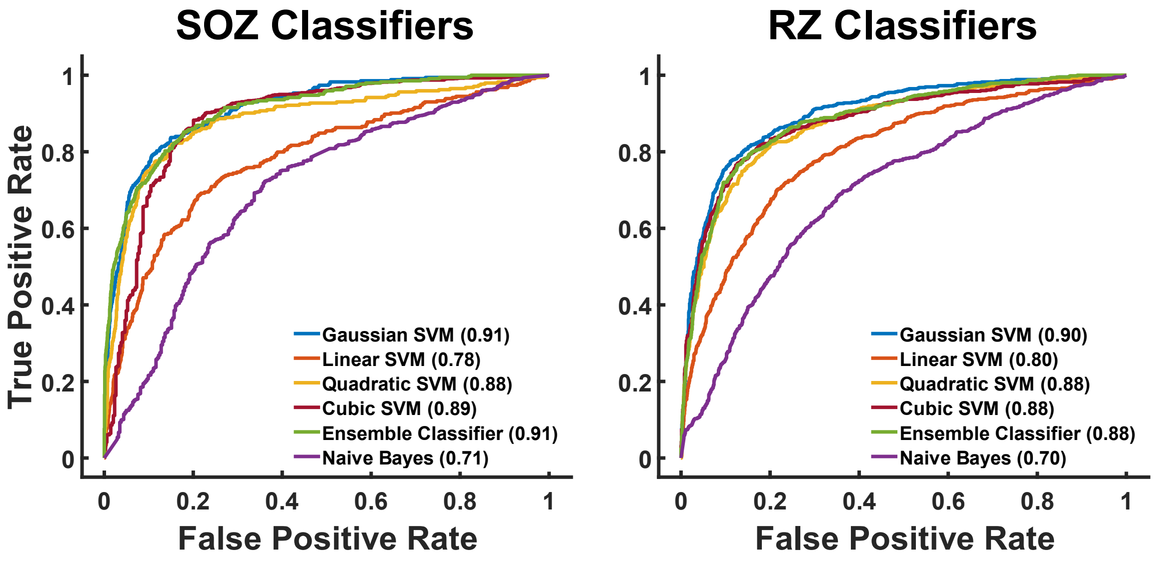

ROC curves for six different classifier types for both SOZ and RZ prediction. All classifiers were trained using the same node level 4-fold partition and all 45 features. The Gaussian Support Vector Machine (SVM), Cubic SVM, and Ensemble classifiers were used for comparisons of performance with random feature selections.

Figure S2

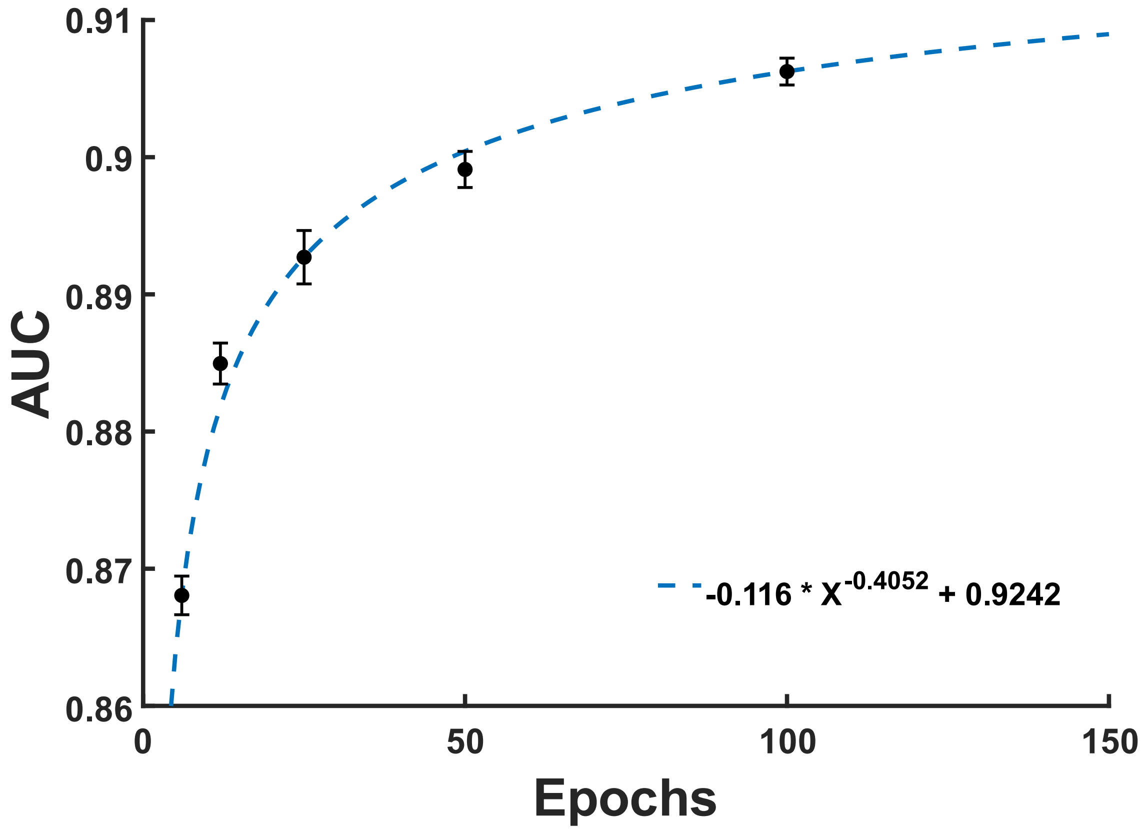

Classification performance as a function of the number of epochs used (out of 150 available) for feature extraction. Gaussian SVM classifiers were trained using all 45 features as inputs. Points represent mean AUC values across random selections and error bars represent standard errors. The fit line is a power fit.

Figure S3

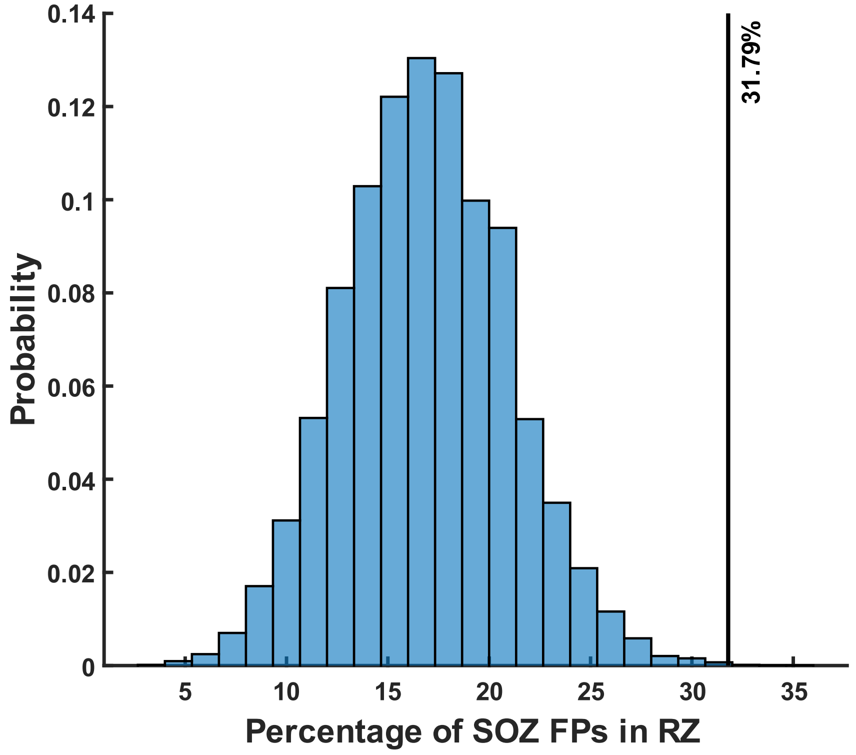

Permutation test distribution of false positives (FPs) from the trained SOZ classifier which fall within the clinically annotated RZ. The solid line denotes the true percentage of SOZ false positives from the trained classifier that fall within the RZ. The 31.79% value is significantly higher than what would be expected from a spatially random distribution of SOZ classifier FP channels as defined by the permutation test distribution (Z-test, Z = 4.028, p = 5.62E-05).

Figure S4

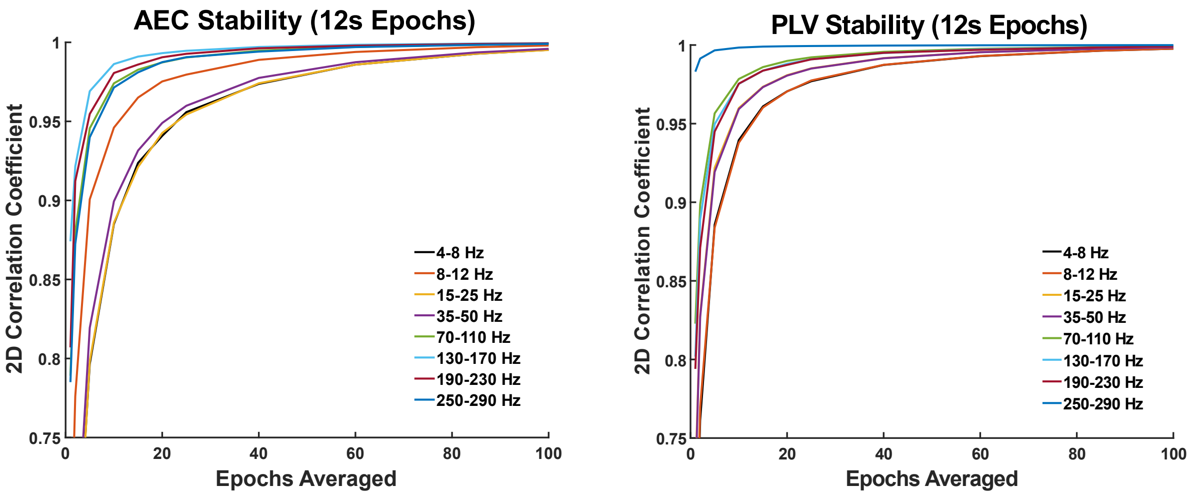

FC metric stability for the eight frequency bands used to calculate AEC (A) and PLV (B). Tracings show the r values between the functional connectome calculated using the average time course across all 150 epochs versus those calculated using the average time course from a randomly selected subset of epochs. For all frequency bands, r > 0.95 at 25 epochs for AEC and 15 epochs for PLV.
